# Ruptured Sinus of Valsalva Aneurysm: Three Case Reports and Literature Review

**DOI:** 10.7759/cureus.59220

**Published:** 2024-04-28

**Authors:** Natalia Moguillansky, Mark Bleiweis, Jana Reid, Jeffrey P Jacobs, Diego Moguillansky

**Affiliations:** 1 Department of Medicine, Division of Pulmonary Critical Care and Sleep Medicine, University of Florida Health, Gainesville, USA; 2 Department of Pediatrics, Cardiology, Congenital Heart Center, University of Florida Health, Gainesville, USA; 3 Department of Cardiology, Congenital Heart Center, University of Florida Health, Gainesville, USA; 4 Department of Pediatrics, Cardiovascular Medicine and Internal Medicine, Congenital Heart Center, University of Florida Health, Gainesville, USA

**Keywords:** ventricular septal defect (vsd), mri cardiac, cardiac mri, transesophageal echocardiography (tee), sinus of valsalva aneurysm

## Abstract

Sinus of Valsalva aneurysm rupture (SOVAR) into the right cardiac chambers is an uncommon complication with unusual presentation, high morbidity and mortality, and unique hemodynamics as well as cardiac imaging findings. Here, we present three SOVAR cases (two with rupture into the right atrium and one with rupture into the right ventricle) that were initially confused for ventricular septal defects and describe their initial presentation, cardiac imaging studies, invasive hemodynamics, as well as treatment options. Some of the unique findings of SOVAR patients include an acute presentation, often with hemodynamic decompensation, the presence of a continuous murmur on examination, and also hemodynamics that include wide pulse pressure and right heart volume overload.

## Introduction

Sinus of Valsalva aneurysm, an enlargement of the aortic root area between the aortic valve annulus and the sinotubular ridge, is an uncommon congenital or acquired cardiac defect that presents in approximately 0.09% of the general population [1]. Several complications can arise, including rupture. Signs and symptoms of sinus of Valsalva rupture (SOVAR) are variable depending on the chamber into which the SOVAR ruptures, typically to the right atrium (RA) or right ventricle (RV) or less common to the left ventricle (LV). The presentation is usually acute, with new-onset shortness of breath and a continuous heart murmur in a previously asymptomatic patient. Echocardiography (transthoracic and transesophageal), magnetic resonance imaging (MRI), cardiac catheterization, and cardiac computed tomography (CT) are modalities used to diagnose it. SOVAR is, unfortunately, often mistaken for a ventricular septal defect (VSD) due to the presence of a murmur and the location of the defect. SOVAR rupture into the RV is particularly confused for a perimembranous VSD (when the non-coronary sinus ruptures into the RV) or an outlet VSD (when the right coronary sinus ruptures into the RV), and rupture into the RA for a Gerbode-type VSD. We present three cases of SOVAR and describe the unique clinical and cardiac imaging characteristics, along with a review of the most recent literature. 

## Case presentation

Case 1 

A 29-year-old male with no significant past medical history was referred to our outpatient clinic for evaluation of a suspected VSD. He complained of dyspnea with minimal exertion and lower extremity edema for six months before presentation. He was initially evaluated at an outside hospital (OSH), where he was treated with diuretics and had a transthoracic echocardiogram (TTE) and a heart catheterization with an initial diagnosis of a VSD.

On our physical examination, his heart rate was 126 beats per minute, his blood pressure was 150/80 mmHg, his oxygen saturation was 100% on room air, and his body mass index was 30. Cardiac examination revealed a III/VI continuous murmur best heard at the mid-sternal border. No rubs or gallops were noted. He had mild edema in the lower extremities. The rest of the examination was normal. 

**Figure 1:**
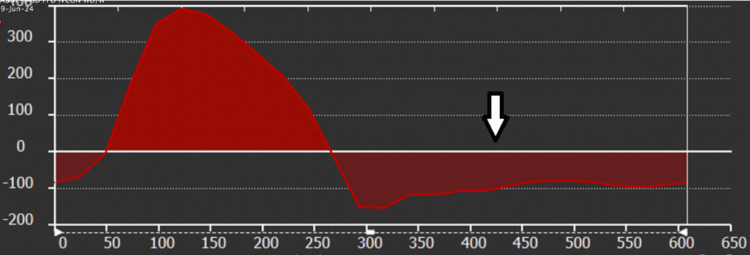
Cardiac Magnetic Resonance Imaging. Aortic flow analysis shows a very large aortic regurgitant fraction of 70% (the arrow shows reverse aortic flow). In the absence of significant visual aortic valve insufficiency, this reflects large left-to-right flow across the SOVAR. The arrow shows the aortic flow below the line, which is the regurgitant or backward flow. The flow above the line is the forward flow. The ratio between them is the aortic valve-calculated regurgitant fraction.

**Figure 2:**
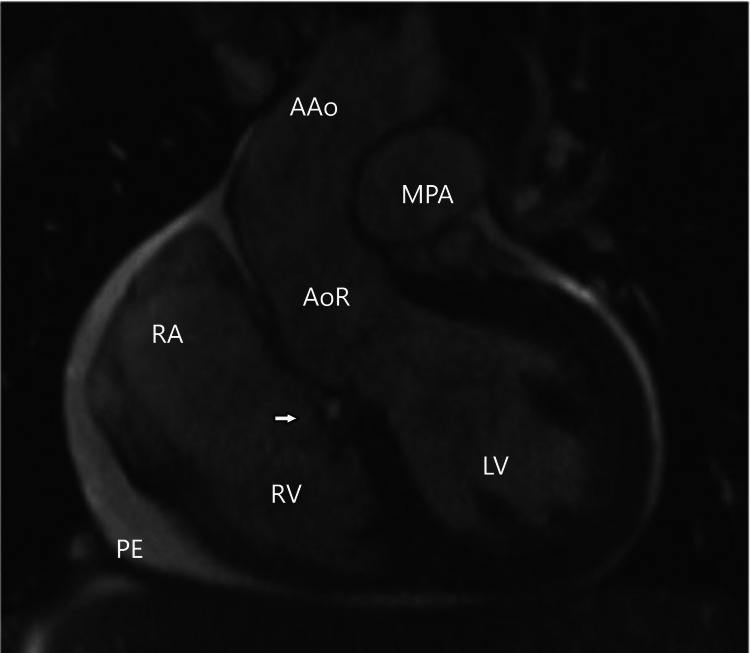
Cardiac Magnetic Resonance Imaging. Still frame of a steady-state free precession (SSFP) left ventricle-aortic root cine, showing a SOVAR between the non-coronary aortic sinus and the right ventricle (arrow), with the jet entering the right ventricle just distal to the plane of the tricuspid valve. A circumferential pericardial effusion is also noted. PE: pericardial effusion; RA: right atrium; RV: right ventricle; LV: left ventricle; AoR: aortic root; AAo: ascending aorta; MPA: main pulmonary artery.

An electrocardiogram (ECG) at our institution showed sinus tachycardia with a normal axis and non-specific repolarization changes. TTE showed a mildly dilated LV with moderately to severely decreased LV systolic function (ejection fraction (EF) 25%-35%) and a moderate-size defect communicating the aortic root and the right ventricle. The differential diagnosis included a perimembranous VSD or a SOVAR into the RV. The presence of continuous flow on Doppler interrogation of the jet between the aortic root and the RV was more consistent with a SOVAR. A cardiac magnetic resonance imaging (MRI) scan showed moderate RV dilatation (end-diastolic volume (EDV) index of 145 ml/m2), severe RV dysfunction (EF 25%), mild LV dilatation (EDV index 117 ml/m2) with global hypokinesis, and moderate to severe LV dysfunction (EF 33%). An interesting MRI finding was the presence of severe calculated aortic regurgitation (regurgitant fraction 70% by phase contrast) without significant visual aortic insufficiency, reflecting large left-to-right shunting across the SOVAR into the RV (Figures 1, 2, and Video 1).

**Video 1:** Cardiac Magnetic Resonance Imaging. Steady-state free precession (SSFP) left ventricular-aortic root cine, showing a SOVAR between the non-coronary aortic sinus and the right ventricle, with the jet entering the right ventricle just distal to the plane of the tricuspid valve. A circumferential pericardial effusion as well as poor biventricular systolic function are also noted. No significant aortic valve insufficiency was seen, despite the calculated large aortic regurgitant fraction (SOVAR-C1-5).

**Figure 3:**
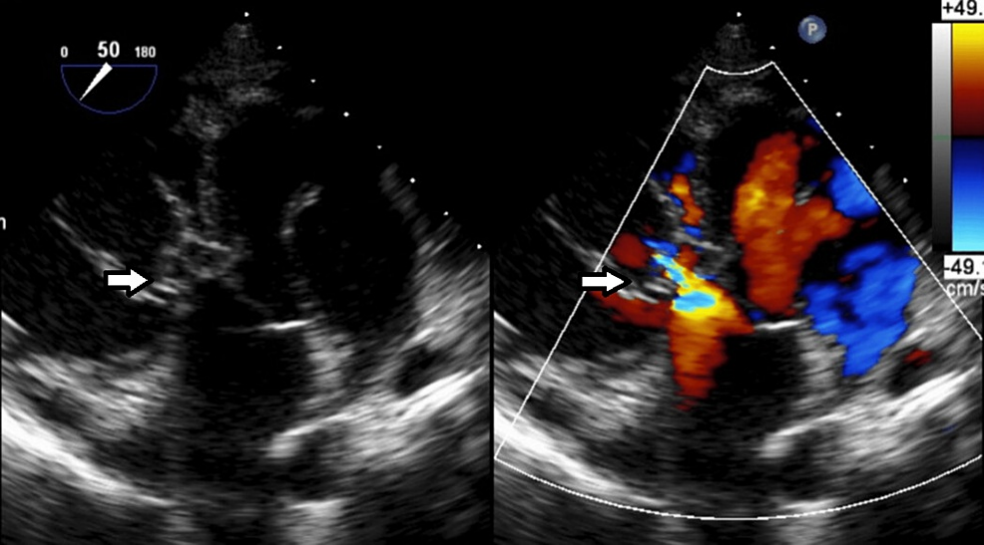
Transesophageal Echocardiogram (TEE). Deep transgastric TEE color-compare still frame showing a ruptured non-coronary sinus of Valsalva aneurysm into the right ventricle with left-to-right shunting (arrow).

**Figure 4:**
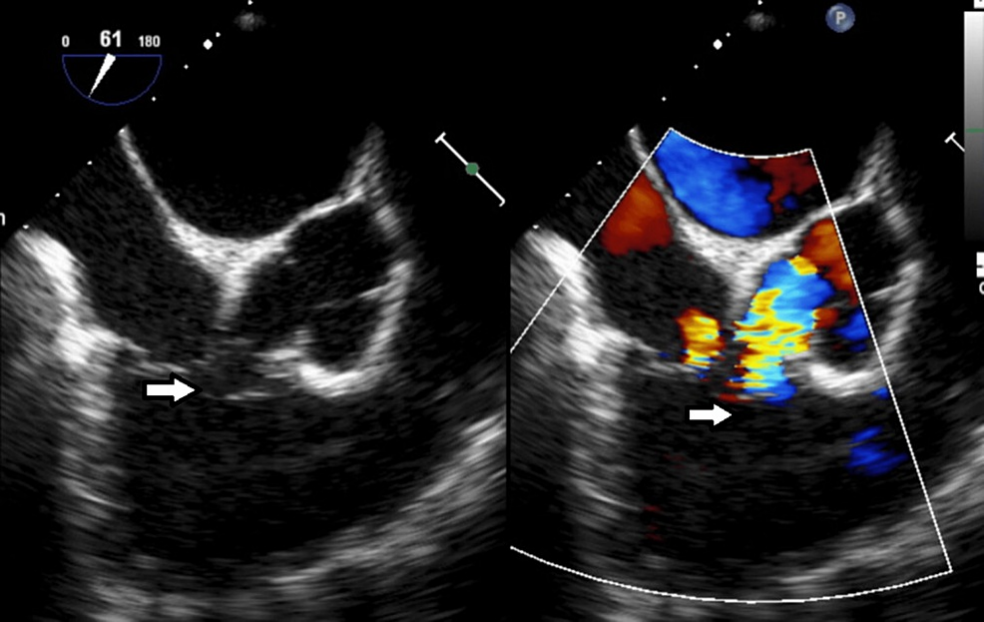
Transesophageal Echocardiogram (TEE). Mid-esophageal TEE color-compare still frame showing a short axis of the aortic valve and aortic root, with a ruptured non-coronary sinus of Valsalva aneurysm into the right ventricle with left-to-right shunting. The aorta-to-right ventricular jet enters the right ventricle just distal to the plane of the tricuspid valve. On the left (2D image without color), the arrow shows the SOVAR into the right ventricle. The right (color image) arrow shows the SOVAR shunting jet entering the right ventricle.

**Figure 5:**
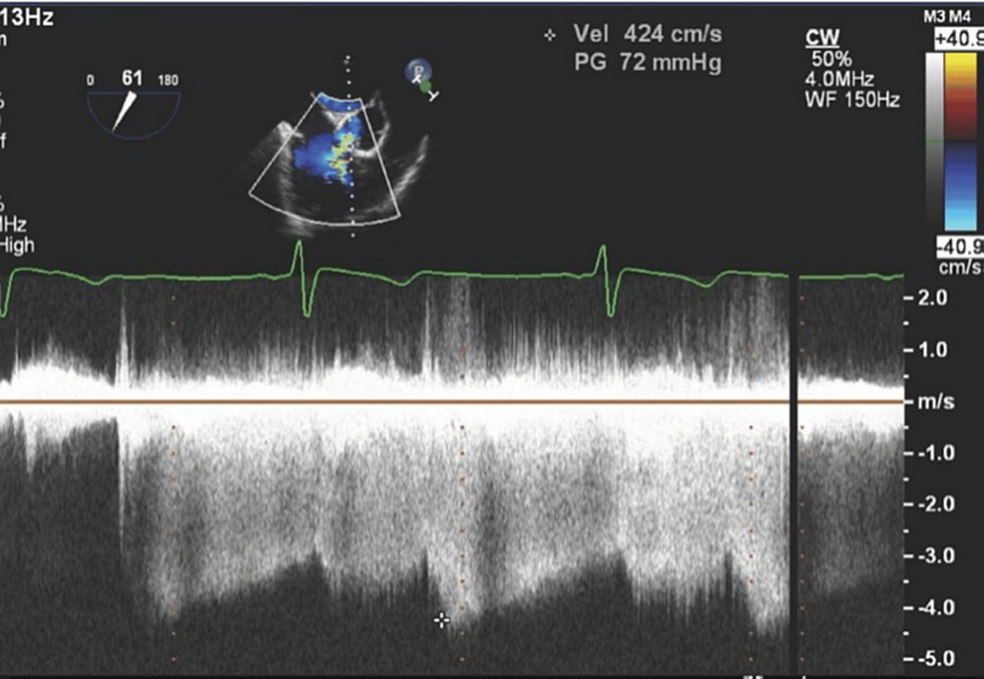
Transesophageal Echocardiogram (TEE). Mid-esophageal TEE still frame showing continuous doppler analysis of the left-to-right aorta-to-right ventricle shunt seen in SOVAR-C1-2. Doppler shows a continuous left-to-right shunt (below the line). A high pressure gradient (peak velocity over 4.2 m/s, consistent with a peak gradient across the defect grater of 72 mmHg) suggests normal right ventricular pressures.

On the first visit, he was not felt to be an adequate surgical candidate due to decompensated heart failure and biventricular systolic dysfunction. For this reason, he was started on medical therapy with diuretics, beta-blockers, and angiotensin-converting enzyme inhibitors (ACEIs). He had significant improvement in his symptoms with resolution of his edema, improvement in his shortness of breath, as well as normalization of his LV and RV systolic function. After optimization of his clinical presentation and ventricular function, we obtained a cardiac catheterization and a transesophageal echocardiogram (TEE) in preparation for surgical repair. Transesophageal (TEE) confirmed the finding of a SOVAR with a large shunt with continuous flow between the noncoronary sinus of Valsalva and the RV (Figures 3-5 and Videos 2, 3). Cardiac catheterization showed normal coronary arteries, elevated RV (50/12 mmHg) and pulmonary artery (PA) pressures (55/22, mean 33 mmHg), normal cardiac output (5.2 L/min, 2.8 L/min/m2), normal pulmonary vascular resistance (PVR) of 1.7 Wood Units (WU), and large left-to-right shunt with a pulmonary to systemic flow ratio (Qp/Qs ratio) of 2.1:1. Wide pulse pressure (100/40 mmHg) with low diastolic blood pressure due to aortic diastolic run-off, without visual aortic insufficiency by TEE, was also noticeable in his catheterization hemodynamics (Figures 3-5 and Videos 2, 3).

**Video 2:** Transesophageal Echocardiogram (TEE). Deep transgastric TEE color-compare movie showing a ruptured non-coronary sinus of Valsalva aneurysm into the right ventricle with left-to-right shunting.

**Video 3:** Transesophageal Echocardiogram (TEE). Mid-esophageal TEE color-compare movie showing a short axis of the aortic valve and aortic root, with a ruptured non-coronary sinus of Valsalva aneurysm into the right ventricle with left-to-right shunting. The aorta-to-right ventricular jet enters the right ventricle just distal to the plane of the tricuspid valve.

Eight months after his initial presentation to our center, he underwent surgical repair of his SOVAR. After initiation of cardiopulmonary bypass with bicaval cannulation and ascending aorta cannulation, the aorta was opened transversally just above the sinotubular junction, confirming the defect between the non-coronary sinus and the right ventricle from a ruptured non-coronary-sinus-of-Valsalva aneurysm. The defect was then closed with a single patch made of Photo-Fix bovine pericardium and 4.0 Prolene sutures.

His postoperative echocardiogram showed no evidence of residual shunting lesions with preserved LV and RV systolic function and no significant valvular disease. Outpatient cardiology follow-up at one, three, and six months after hospital discharge showed good results with no residual shunting, normal ventricular function, and no active cardiac symptoms.

Case 2 

A 34-year-old male with no significant prior cardiac history was hospitalized for an OSH due to a two-month history of progressive shortness of breath associated with peripheral edema, pulmonary edema, and ascites. Before the hospitalization, he had been active and asymptomatic. He was started on hemodialysis (HD) due to acute oliguric renal failure with large volume overload. After his initial TTE at the OSH, which revealed what was thought to be a VSD, he was transferred to our center for further evaluation and management. On arrival at our institution, his examination showed a blood pressure of 113/47 mmHg, a respiratory rate of 16, a pulse of 82 beats per minute, and a body mass index of 28. Initial oxygen requirements at the OSH had resolved on arrival after he had had volume removed with HD, and he was able to maintain pulse oximetry>95% on room air. He had a III/VI continuous murmur at the left sternal border. He also had anasarca, with ascites, peripheral edema, and bibasilar crackles on lung examination. Upon arrival, he was also making urine despite HD, so he was started on a furosemide drip with a good response, and HD was able to be discontinued.

**Figure 6:**
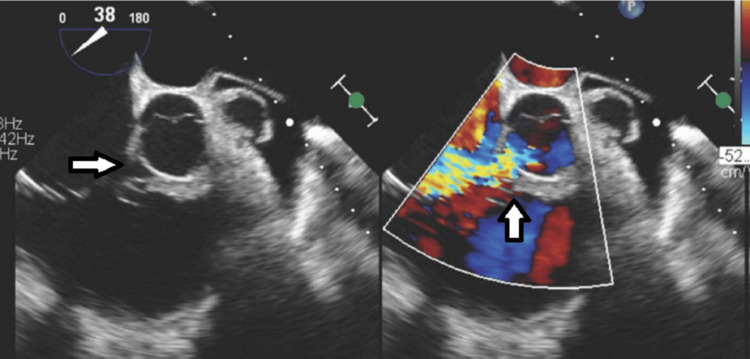
Transesophageal Echocardiogram (TEE). Mid-esophageal TEE color-compare still frame showing a short axis of the aortic valve and aortic root, with a ruptured non-coronary sinus of Valsalva aneurysm into the right atrium with left-to-right shunting (left arrow). The aorta-to-right atrium jet enters the right atrium just above the plane of the tricuspid valve leaflets (right arrow).

**Figure 7:**
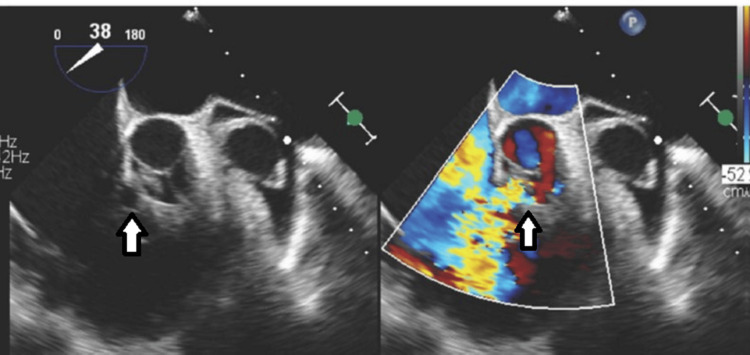
Transesophageal Echocardiogram (TEE). Mid-esophageal TEE color-compare still frame showing a short axis of the aortic valve and aortic root, with a ruptured non-coronary sinus of Valsalva aneurysm into the right atrium with left-to-right shunting (left arrow). The aorta-to-right atrium jet enters the right atrium just above the plane of the tricuspid valve leaflets and then enters the right ventricle across the tricuspid valve leaflet opening (right arrow).

**Figure 8:**
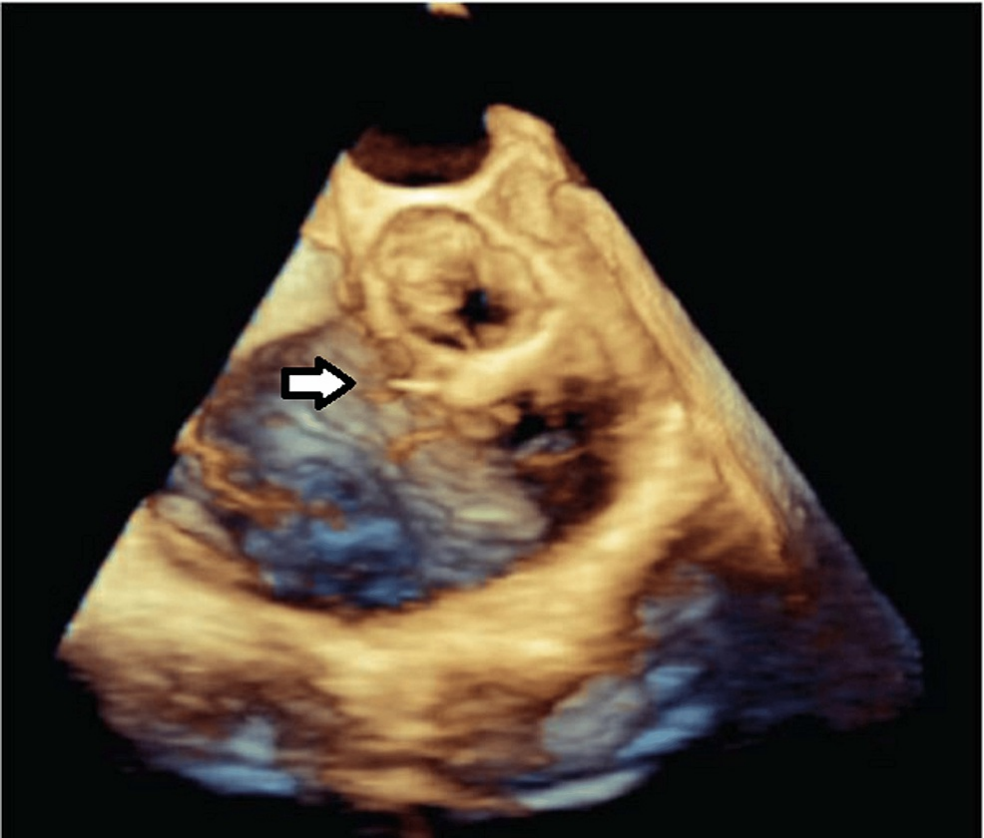
Transesophageal Echocardiogram (TEE). Mid-esophageal TEE 3D still frame showing the aortic valve and root, the tricuspid valve leaflets, and the SOVAR between the non-coronary sinus of the aortic root and the right atrium, providing a 3D view of the defect (the arrow shows the SOVAR).

**Figure 9:**
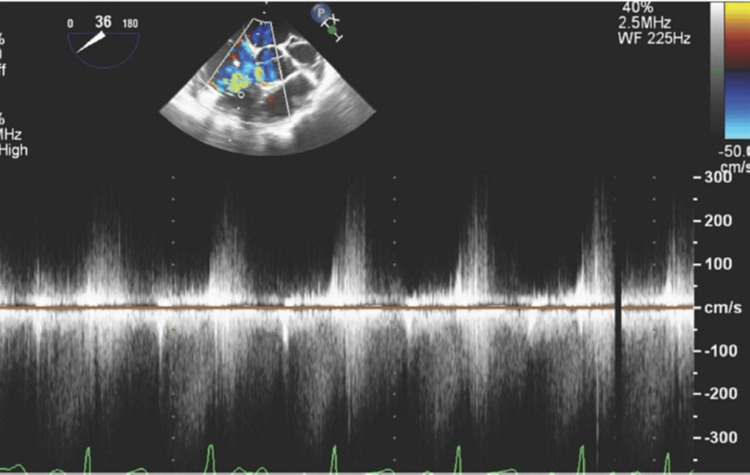
Transesophageal Echocardiogram (TEE). Mid-esophageal TEE still frame showing continuous doppler analysis of the left-to-right aorta-to-right atrium shunt seen in SOVAR. Doppler shows a continuous left-to-right shunt (continuous flow below the line).

**Figure 10:**
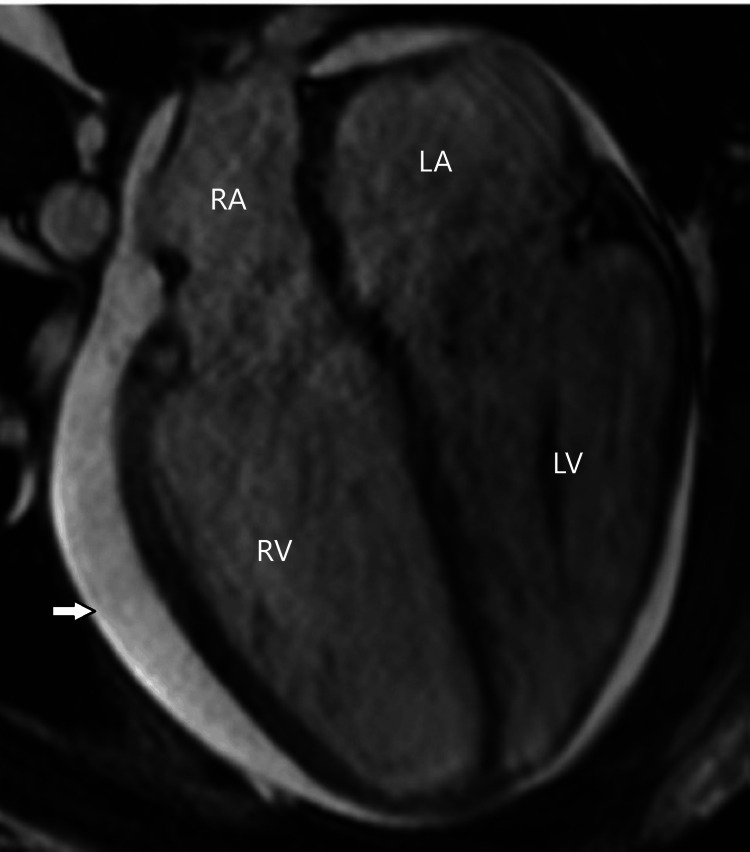
Cardiac Magnetic Resonance Imaging (MRI). Still frame of a four-chamber steady-state free precession (SSFP) cine MRI showing normal left ventricular size, mild right ventricular dilation, and small to moderate size pericardial effusion (arrow). RA: right atrium; RV: right ventricle; LA: left atrium; LV: left ventricle

**Figure 11:**
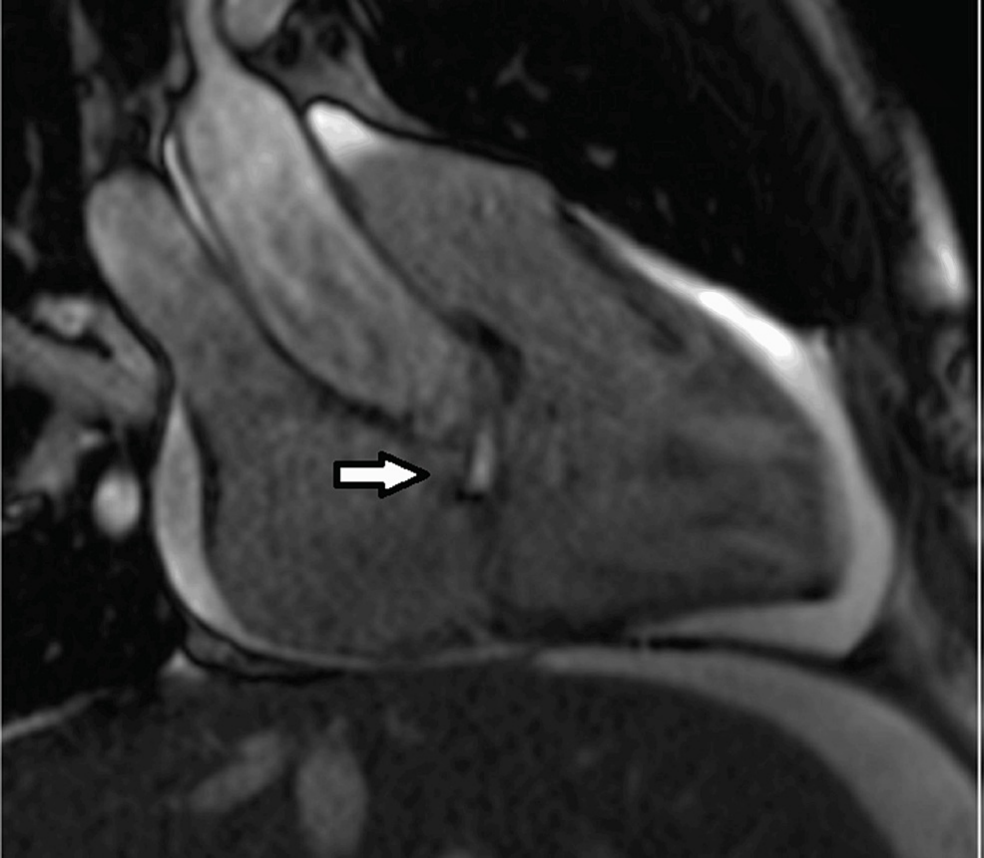
Cardiac Magnetic Resonance Imaging (MRI). Still frame of a modified right ventricular inflow-outflow steady-state free precession (SSFP) cine MRI, including the aortic root and showing a SOVAR into the right atrium just above the plane of the tricuspid valve (arrow shows the jet entering the right atrium).

**Figure 12:**
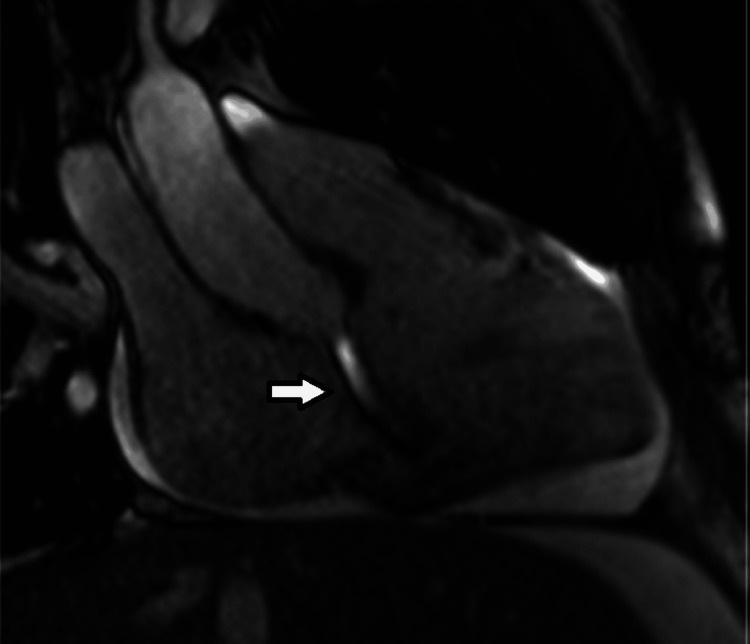
Cardiac Magnetic Resonance Imaging (MRI). Still frame of a modified right ventricular inflow-outflow steady-state free precession (SSFP) cine MRI, including the aortic root, obtained in the cardiac cycle immediately after the image obtained in SOVAR-C2-2, showing the shunt starts in the aortic root, then enters the right atrium just above the plane of the tricuspid valve, and then enters the right ventricle through the tricuspid valve opening (arrow shows the SOVAR jet entering the right ventricle in forward fashion through the tricuspid valve leaflet opening).

**Figure 13:**
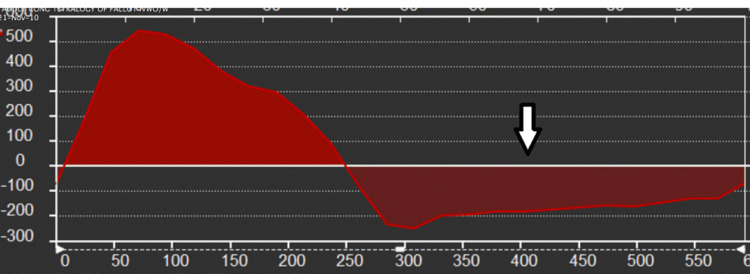
Cardiac Magnetic Resonance Imaging. Aortic flow analysis shows a very large aortic regurgitant fraction of 71%. In the absence of significant visual aortic valve insufficiency, this reflects large left-to-right flow across the SOVAR. Arrows highlight the portion of the aortic flow curve below the line, which represents the regurgitant flow. The flow above the line is the forward flow, and the ratio between both provides the aortic valve regurgitant fraction.

Initial TTE showed mild LV dilation with normal systolic function (EF 60%), moderate RV dilation with mildly reduced RV systolic function, and a moderate size defect from the aortic root with flow into the RA, consistent with either a Gerbode-type VSD or more likely a SOVAR into the RA given continuous shunt on doppler analysis (Figures 6-9 and Videos 4, 5). A cardiac MRI was then obtained showing normal LV size (EDV index 96 ml/m2) and systolic function (EF 65%), mildly dilated RV (EDV index 118 ml/m2) with borderline RV systolic function (EF 48%), and a SOVAR (non-coronary sinus rupture) into the RA and through the tricuspid valve into the RV as well, with significant left-to-right shunting (Figures 10-13 and Videos 6-8). MRI calculation of the Qp/Qs ratio could not be adequately performed by comparison of aortic and pulmonary artery flows, but the presence of a calculated aortic valve regurgitation fraction of 71% with no visual aortic insufficiency was consistent with a large left-to-right shunt, as ~71% of the LV stroke volume was being shunted to the right heart. As a result of left-to-right shunting through the ruptured sinus of the Valsalva, there was markedly reduced aortic forward flow (only 24 ml/beat). Inotropic support with dobutamine (3-5 mcg/kg/min) was added to the furosemide infusion, with improvement in symptoms, edema, and eventually renal function as well. TEE confirmed the presence of a moderate-size (orifice 1.3 cm) SOVAR from the aortic root non-coronary sinus into the RA with continuous left-to-right flow. After diuresis and inotropic support, cardiac catheterization showed SOVAR into the RA with a Qp/Qs ratio of 1.9:1, RA pressure of 17 mmHg, RV pressure of 45/19 mmHg, PA pressure of 42/25 mmHg with a mean of 31 mmHg, and a PA wedge and LVEDP both of 24 mmHg. Cardiac output was low despite inotropic support (4 L/min and 2.06 L/min/m2) with normal PVR (0.93 WU) and normal coronary arteries on angiography. 

**Video 4:** Transesophageal Echocardiogram (TEE) Mid-esophageal TEE color-compare movie showing a short axis of the aortic valve and aortic root, with a ruptured non-coronary sinus of Valsalva aneurysm into the right atrium with left-to-right shunting. The aorta-to-right atrium jet enters the right atrium just above the plane of the tricuspid valve leaflets and then enters the right ventricle across the tricuspid valve leaflet opening.

**Video 5:** Transesophageal Echocardiogram (TEE). Mid-esophageal TEE 3D movie showing the aortic valve and root, the tricuspid valve leaflets, and the SOVAR between the non-coronary sinus of the aortic root and the right atrium, providing a 3D view of the defect.

**Video 6:** Cardiac Magnetic Resonance Imaging (MRI). Four-chamber steady-state free precession (SSFP) cine MRI showing normal left ventricular size and systolic function, mild right atrium and right ventricular dilation with borderline right ventricular systolic function, and a small-to-moderate size pericardial effusion.

**Video 7:** Cardiac Magnetic Resonance Imaging (MRI). Left ventricular-aortic root steady-state free precession (SSFP) cine MRI, including the right atrium, shows the shunt starts in the aortic root and enters the right atrium just above the plane of the tricuspid valve.

**Video 8:** Cardiac Magnetic Resonance Imaging (MRI). Modified RV inflow-outflow steady-state free precession (SSFP) cine MRI, including the right atrium-right ventricular outflow tract and the aortic root, showing the SOVAR shunt starts in the aortic root, then enters the right atrium just above the plane of the tricuspid valve, and then enters the right ventricle through the tricuspid valve opening.

After medical optimization, he underwent SOVAR surgical repair 22 days after his initial presentation. After initiation of cardiopulmonary bypass with bicaval cannulation and ascending aorta cannulation, the right atrium was initially opened to attempt a transatrial approach. Through the right atrium, the defect was well visualized, confirming the diagnosis of a ruptured non-coronary sinus of Valsalva aneurysm into the right atrium. Due to excessive blood in the field, the procedure was switched to a trans-aortic approach. The aorta was opened transversally just above the sinotubular junction, again confirming the defect between the non-coronary sinus and the right atrium from a ruptured non-coronary sinus of Valsalva aneurysm. The defect was then closed with a single patch made of Photo-Fix bovine pericardium and 5.0 Prolene sutures. The aortotomy site was also closed with 5.0 Prolene sutures.

The post-operative echocardiogram showed no residual SOVAR and preserved biventricular systolic function. During hospitalization, his renal function completely recovered, hemodialysis was discontinued, and he was discharged home two weeks after surgical repair. One month after hospital discharge, he was seen as an outpatient when he had resolved all his symptoms. His anasarca and signs of heart failure had also resolved. 

Case 3 

A 24-year-old male without known medical problems was hospitalized following a motor vehicle accident for 10 days due to splenic and liver laceration, as well as traumatic hemorrhagic pericardial tamponade. He underwent chest and abdominal exploration with surgical evacuation of the tamponade, as well as repair of the liver and splenic lacerations. Intraoperative TEE showed significant tricuspid regurgitation (TR) that was thought to be a perimembranous VSD. He recovered and was discharged home without any cardiac interventions. Subsequently, his primary care physician noted a murmur on physical examination and obtained a TTE showing mild right atrium and ventricular dilation, normal LV and RV systolic function, severe tricuspid regurgitation, and again what was thought to be a VSD. He denied any episodes of chest pain, shortness of breath, edema, palpitations, or syncope. He was not particularly active but described no exercise limitations. 

**Figure 14:**
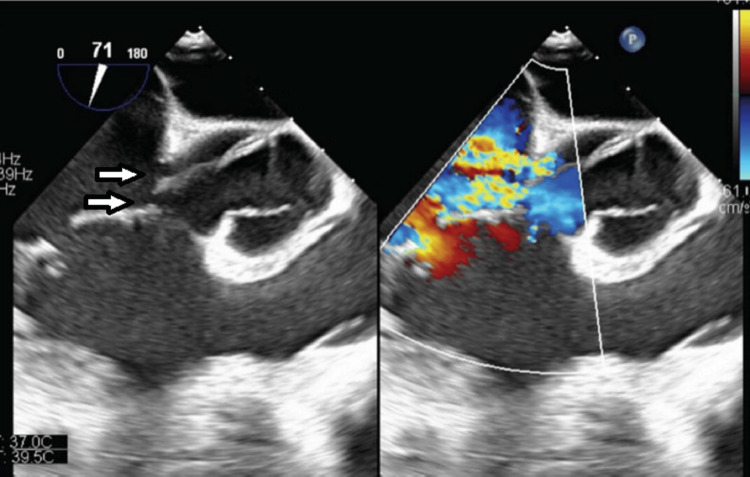
Transesophageal Echocardiogram (TEE). Mid-esophageal TEE color-compare still frame showing a short axis of the aortic valve and aortic root, with a ruptured non-coronary sinus of Valsalva aneurysm into the right atrium with left-to-right shunting from two separate defects (arrows). The aorta-to-right atrium jet enters the right atrium just above the plane of the tricuspid valve leaflets.

He was seen at our institution three months after his motor vehicle accident. His ECG showed a normal sinus rhythm. TTE and TEE showed SOVAR from the non-coronary sinus to the RA, as well as a flail anterior tricuspid valve leaflet with moderate to severe tricuspid regurgitation. The RV was mildly dilated with normal systolic function, and the LV size and systolic function were normal (Figure 14 and Video 9). Cardiac catheterization showed SOVAR into the RA with a mild to moderate left-to-right shunt and a Qp/Qs ratio of 1.4:1. Mildly elevated right-sided pressures were noted (RA mean 13 mmHg with a V wave of 17 mmHg, RV mean 32/14 mmHg, PA mean 32/15 mmHg with a mean of 22 mmHg), with normal PA wedge and LVEDP (13 and 12 mmHg, respectively), mildly reduced cardiac output (4 L/min and 2.2 L/min/m2), and normal PVR (1.55 WU).

**Video 9:** Transesophageal Echocardiogram (TEE). Mid-esophageal TEE color-compare movie showing a short axis of the aortic valve and aortic root, with a ruptured non-coronary sinus of Valsalva aneurysm into the right atrium with left-to-right shunting from two separate defects. The aorta-to-right atrium jet enters the right atrium just above the plane of the tricuspid valve leaflets.

Three months after presentation, he underwent surgical repair with the closure of the SOVAR, as well as repair of the tricuspid valve with anterior leaflet plication and annuloplasty. The surgical approach included aortic and bicaval cannulation and cardiopulmonary bypass. A right atrial atriotomy was performed, allowing visualization of a defect between the non-coronary aortic sinus and the right atrium. The defect was closed with a single patch made of Photo-Fix bovine pericardium. Attention was then switched to the flail tricuspid leaflet, which was repaired with 5.0 Prolene sutures.

Post-operative TTE showed no residual shunting defects and only mild residual tricuspid regurgitation. He has remained asymptomatic in his outpatient follow-up for two years after surgery. 

## Discussion

**Figure 15:**
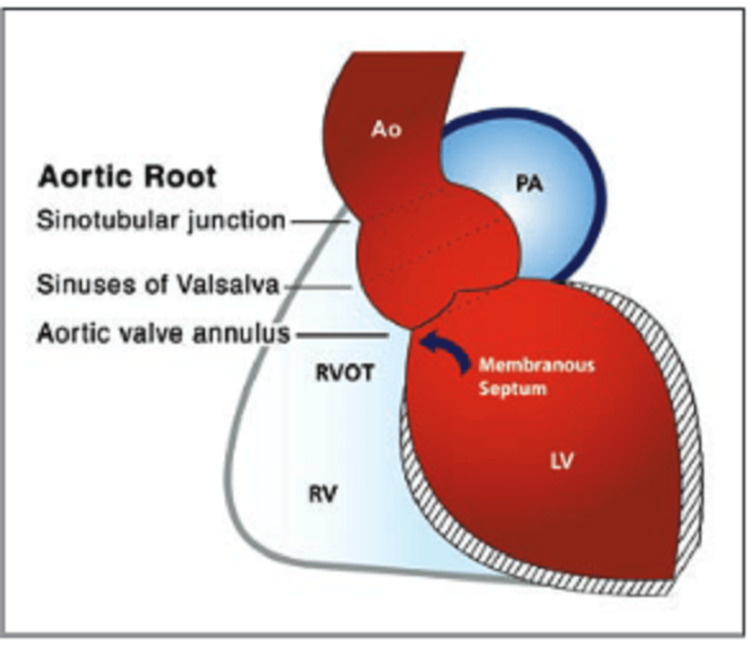
A diagrammatic representation of the aortic root in the coronal oblique plane illustrates normal anatomy and relationships. Ao: Aorta, PA: Pulmonary artery, RVOT: Right ventricular outflow tract, RV: Right ventricle, LV: Left ventricle. Reprinted with permission from Hoey, E.T., A. Kanagasingam, and M.U. Sivananthan, Sinus of Valsalva aneurysms: assessment with cardiovascular MRI. AJR Am J Roentgenol, 2010. 194(6): p. W495-504.

**Figure 16:**
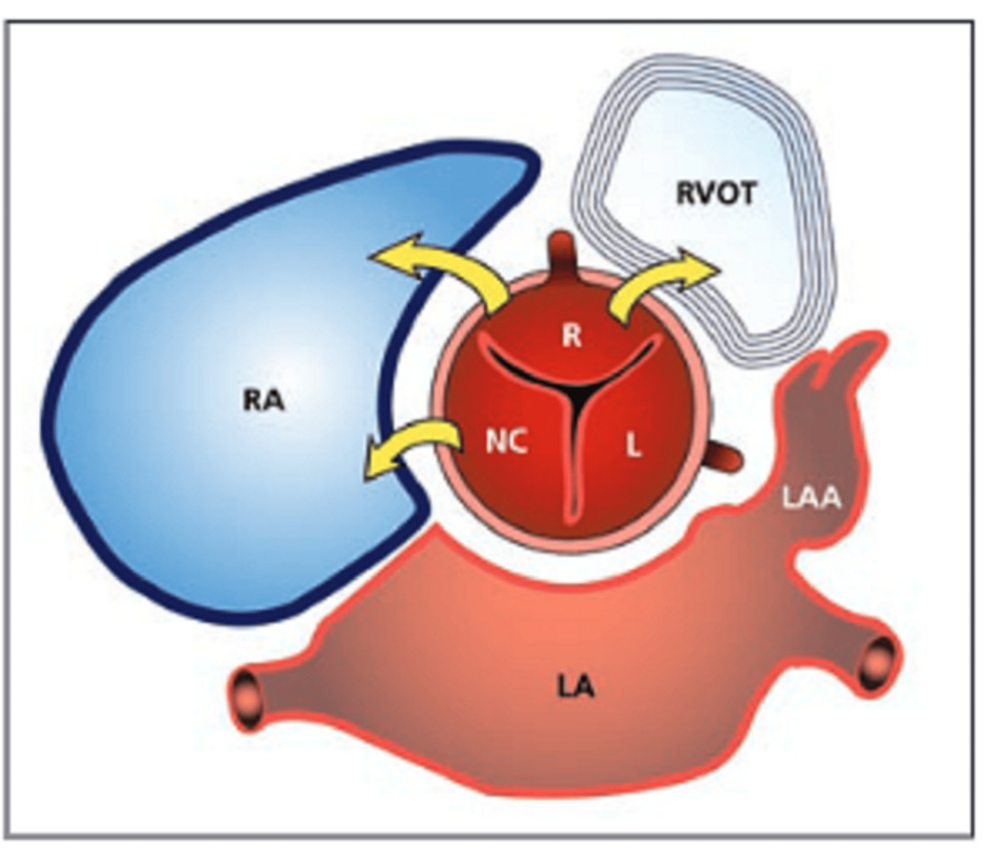
A diagrammatic representation of the sinus of Valsalva illustrates its relationship with adjacent cardiac chambers and the most frequent sites of rupture. RA: Right atrium, RVOT: Right ventricular outflow tract, LA: Left atrium, LAA: Left atrial appendage, R: Right cornary cusp, L: Left coronary cusp, and NC: Non-coronary cusp. Reprinted with permission from Hoey, E.T., A. Kanagasingam, and M.U. Sivananthan, Sinus of valsalva aneurysms: assessment with cardiovascular MRI. AJR Am J Roentgenol, 2010. 194(6): p. W495-504.

There are normally three aortic sinuses. The left and right sinuses each contain their respective coronary artery ostias, whereas the posterior sinus is the noncoronary sinus. The normal sinus diameter is 4 cm for men and 3.6 cm for women. The sinus of Valsalva aneurysm is an enlargement of the aortic root area between the valve annulus and the sinotubular junction [2,3] (Figures 15, 16). An analysis of 86 patients by Mustafa et al. in 2007 showed that the aneurysms arose from the right coronary sinus in 70% of the cases, the non-coronary sinus in 25%, and the left coronary sinus in 5% [4].

Sinus of Valsalva aneurysm is a rare condition with an estimated prevalence of 0.09% of the general population and 0.1 to 3.5% of all congenital cardiac defects [5]. Sinus of Valsalva aneurysms can be congenital or acquired. Congenital aneurysms are associated with Marfan syndrome, Ehlers-Danlos syndrome, or other connective tissue disorders. Acquired aneurysms have been associated with trauma, atherosclerosis, infective endocarditis, iatrogenic injury during aortic valve replacement, syphilis, and collagen vascular disorders [6]. Complications of sinus Valsalva aneurysms include rupture, aortic regurgitation, arrhythmia, outflow obstruction, myocardial ischemia, endocarditis, and iatrogenic embolism [3]. 

The clinical presentation of SOVAR is characterized by previously healthy individuals between the ages of 20 and 40 who develop a loud, machinery-like, continuous murmur during systole and diastole, with the best heart at the base of the heart [7]. There are two clinical patterns of symptoms of SOVAR: the acute rupture of a large Sinus of Valsalva aneurysm and the gradual progression of a small perforation. The first pattern presents with acute dyspnea, marked substernal chest pain, and possible hemodynamic collapse. Progressive dyspnea, fatigue, orthopnea, and paroxysmal nocturnal dyspnea can all occur after the acute rupture. Sudden cardiac death may result from both ruptured and unruptured aneurysms [1].

Ruptured aneurysms originate most frequently from the right coronary sinus (65-85%), less frequently from the non-coronary sinus (10-30%), and rarely from the left coronary sinus (<5%). The right ventricle is the most common receiving chamber (about 80-90%) [1].

The initial diagnostic modalities to evaluate SOVAR are usually TTE and TEE. Evaluation with TTE/TEE will reveal a continuous flow in systole and diastole in SOVAR in about 70% of the cases [8,9], and this is one of the hallmarks of the diagnosis.

Axial imaging with cardiac MRI or CT is very helpful to provide a 3D view of the defect and confirm the diagnosis. Cardiac MRI can provide additional information, including RV/LV volumes and systolic function, visualization of the defect in multiple planes, magnitude of shunting, and the presence of associated conditions.

Cardiac catheterization can be used to confirm the diagnosis and further evaluate the hemodynamic significance of the SOVAR, including the magnitude of shunting, filling pressures, cardiac output, and PVR.

The asymptomatic, unruptured sinus of Valsalva has an unknown natural history, and its management is unclear [10]. Surgical intervention is usually indicated with progressive dilatation or when unruptured aneurysms cause malignant arrhythmia, ostial coronary artery occlusion, or right ventricular outflow tract obstruction. SOVAR requires early surgical intervention since median survival is only 3.9 years in untreated patients, usually with progressive symptoms and clinical decompensation [6,11].

Surgical treatment of SOVAR is usually recommended, although percutaneous transcatheter closure has been described [12]. Galeczka et al. described the cases of 23 patients selected for transcatheter closure of SOVAR with Amplatzer devices. The procedure was deemed successful in 19/23 patients (82.6%). Four procedures were abandoned, and the device was percutaneously retrieved due to coronary artery compression (one patient), transient increase of aortic regurgitation (one patient), or embolization (two patients). A new onset of significant aortic regurgitation was noted in one of the latter patients after device removal. In addition, three patients needed percutaneous re-intervention during follow-up because of a significant residual shunt in one and a late recurrent right sinus Valsalva aneurysm in two patients. Other reported complications include post-operative heart block and distortion of the aortic annulus [13].

Surgical procedures include primary closure, patch repair, or aortic root replacement with or without valve replacement [6].

Our cases

Here, we present three SOVAR cases, including clinical presentation, cardiac imaging, hemodynamic evaluation, and surgical intervention. In all three of the cases, the ruptured sinus was the non-coronary sinus, with rupture into the RA in two cases and rupture into the RV in one case (our first case). Two of the cases occurred after spontaneous rupture, and the third case was traumatic after a motor vehicle accident.

Distinguishing SOVAR vs. VSD

In all three cases, the diagnosis of SOVAR was initially confused with a VSD, highlighting the importance of differentiating the clinical and imaging characteristics of both conditions. The clinical presentation of a SOVAR is usually acute and associated with hemodynamic decompensation, usually in a patient not known to have a murmur or any prior cardiac problems. On the other hand, small or moderate-sized unrepaired VSDs are typically known to patients and their medical providers and are either asymptomatic or mildly symptomatic with dyspnea on exertion but without hemodynamic decompensation. Large VSDs are either closed in infancy or usually develop pulmonary hypertension and are typically not confused with SOVAR as the presentation is very different. The murmur of a SOVAR is typically continuous versus pansystolic on a VSD. The Doppler interrogation of the defect correlates with this, showing a continuous shunt in SOVAR as opposed to systolic shunting in a VSD.

Cardiac imaging for SOVAR

TTE is usually the initial diagnostic study and can provide the location of the defect, the chambers involved in the defect, as well as any associated valvular or ventricular function abnormalities. Doppler analysis can characterize the type of shunt as well (left to right vs. bidirectional, continuous shunt vs. pansystolic).

TEE can provide better image resolution of the defect, with the more thorough characterization of the defect size and the chambers involved, as well as any associated abnormalities, and color and spectral Doppler can be used to characterize the type of defect as well. Finally, 3D TEE can help provide a 3D view of the defect, as was nicely illustrated in our second case.

Cardiac MRI is very versatile and can provide a 3D view of the defect and involved cardiac chambers. It can also provide a lot of additional information to characterize the associated hemodynamics. LV and RV volumes and ventricular function, as well as aortic and pulmonary artery flows, can be used to characterize the magnitude of the shunting and the cardiovascular repercussions of the shunt. As can be seen in our first and second cases, MRI can show which sinus of the aortic root ruptured, what chamber it ruptured into, associated valvular disease, associated LV/RV dilation or dysfunction, and the magnitude of shunting. A very interesting finding of the cardiac MRIs in the first and second cases was the presence of a very large calculated aortic valve regurgitant fraction (70-71%) without significant visual aortic valve insufficiency, reflecting a large left to right shunt with over 70% of the LV stroke volume entering the right heart either into the RA or the RV, leading to simultaneous right heart volume load and reduced effective LV cardiac output.

A cardiac computed tomography angiogram does not provide the versatility and dynamic information provided by an MRI but is a helpful study to provide rapid 3D evaluation of the defect, gross volumetric LV/RV assessment, and, when needed, can also be used for coronary artery delineation before cardiac surgery.

Clinical presentation and hemodynamics of SOVAR

Our first and second cases show spontaneous SOVAR into the RA and RV with acute hemodynamic decompensation. Both display the interesting MRI finding of a large calculated aortic valve regurgitant fraction by phase contrast without significant visual aortic insufficiency either by MRI or by TTE/TEE, reflecting a large shunt across the defect into the right heart. These two patients have very interesting and not previously described hemodynamics, with the combination of acute right heart volume load and reduced left-sided effective cardiac output. Compared to acute aortic regurgitation when patients have wide pulse pressure, relatively preserved LV effective cardiac output, and LV volume load, our patients have wide pulse pressure, reduced LV effective cardiac output, and RV volume load rather than LV volume load. As a result, these patients were more hemodynamically unstable and required more medical optimization before surgical repair.

Our third case was related to traumatic SOVAR, leading to a small to moderate shunt into the RA and a flail tricuspid valve with moderate to TR. As the left to right shunt was relatively modest and the TR was well tolerated, this patient was largely asymptomatic, and we offered him surgery mostly to prevent further right heart dilatation and eventual dysfunction due to the combined right heart volume load from concomitant TR, and SOVAR-related left to right shunt.

## Conclusions

History, a physical exam, and comprehensive cardiac imaging are key to differentiating a SOVAR from a VSD. The absence of a prior murmur or a known VSD, acute decompensation often with profound volume overload, and physical exam findings of a continuous murmur and wide pulse pressure all suggest a SOVAR. Large aortic valve regurgitant fraction on MRI without visual aortic insufficiency, as well as continuous systolic and diastolic flow across the shunt, are also unique characteristics of SOVAR to the right heart. 
